# Supplementary material for: Traditional herbal medicine use doubled the risk of multi-organ dysfunction syndrome in children: A prospective cohort study
Source: PLoS One. 2024 Feb 23;19(2):e0286233. doi: 10.1371/journal.pone.0286233 (PMC10889611; doi:10.1371/journal.pone.0286233)
Supplement: Attachment — Submitted filename: Response to the Reviewers.docx [file pone.0286233.s002.docx]

**Response to the Reviewers**

**Manuscript number:** PONE-D-23-14246

# **Title:** Traditional Herbal Medicine Use Doubled the Risk of Multi-organ Dysfunction Syndrome in a Pediatric Intensive Care Unit in the University of Gondar Comprehensive Specialized Hospital, Northwest Ethiopia: A Prospective Cohort Study.

Authors;

Nahom Worku Teshager: ([nahomworkarm@gmail.com](mailto:nahomworkarm@gmail.com))

Koku Sisay Tamirat: [kokusisay23@gmail.com](mailto:kokusisay23@gmail.com)

Ashenafi Tazebew Amare: [ashenafitazebew1@gmail.com](mailto:ashenafitazebew1@gmail.com)

Mulualem Endeshaw Zeleke: [muleaman45@gmail.com](mailto:muleaman45@gmail.com)

Asefa Admasu Taddese: [adimasuasefa@gmail.com](mailto:adimasuasefa@gmail.com)

We are grateful for the helpful feedback and constructive suggestions by the editors and reviewers that helped us to improve the quality of the manuscript. We also appreciate your acknowledgment of the fact that this is a very important study describing the role of traditional Herbal Medicine intake in the risk of development of MODS. This study tried to show how traditional herbal medicine intake affects the incidence of MODS among pediatric age groups. We carefully responded to all points raised by the editorial board and reviewers and have modified the manuscript accordingly. We have submitted the data as a supporting file as we have no legal restrictions. Since all authors are from a developing country (Ethiopia), we are eligible for waiver of the article processing charge. We hope you will consider our work for publication.

With regards!

Authors.

**Response to Editorial Board:**

Dear editorial Boar,

We have amended the manuscript as to the recommendations and comments you provided us. Thank you for contributing for the improvement of our work. We hope we have addressed all the issues/points you stated.

1. Please ensure that your manuscript meets PLOS ONE's style requirements, including those for file naming. The PLOS ONE style templates can be found at

Response: Thank you for the suggestion. It’s done as per the Plos One’s requirement.

2. Your ethics statement should only appear in the Methods section of your manuscript. If your ethics statement is written in any section besides the Methods, please delete it from any other section.

Response: Corrected.

3. We note that you have indicated that data from this study are available upon request. PLOS only allows data to be available upon request if there are legal or ethical restrictions on sharing data publicly.

Response: We have submitted the data as a supporting file.

4. PLOS requires an ORCID iD for the corresponding author in Editorial Manager on papers submitted after December 6th, 2016. Please ensure that you have an ORCID iD and that it is validated in Editorial Manager. To do this, go to ‘Update my Information’ (in the upper left-hand corner of the main menu), and click on the Fetch/Validate link next to the ORCID field. This will take you to the ORCID site and allow you to create a new iD or authenticate a pre-existing iD in Editorial Manager. Please see the following video for instructions on linking an ORCID iD to your Editorial Manager account: <https://www.youtube.com/watch?v=_xcclfuvtxQ>.

Response: We have included the ORCID iD of the corresponding author.

5. Please ensure that you refer to Figures 3, 4 and 5 in your text as, if accepted, production will need this reference to link the reader to the figure.

Response: Done.

**Response to Reviewer #1**

1. The title seems exaggerated. Although the patients who used THM had a 2.45 (95% CI: 1.29, 4.65 ) risk of developing MODS, the authors are trying to exaggerate the output. Hence it has to be said doubled the occurrence of MODS instead of a threefold increase in risk of MODS.

**Response:** Thank you reviewer for the input. We have adjusted it in the revised manuscript as to your recommendation.

2. In the abstract, under the result section, the last sentence is repeatedly mentioned and has to be deleted. The word keyword has to be written as one word and has to be corrected. **Response:** Thank you reviewer for the input. We have adjusted it in the revised manuscript as to your recommendation.
3. In the material and methods section, the study area description is needed. Information regarding the area coverage of the hospital, patient types attending the hospital has to be provided.

**Response:** Thank you reviewer. We have included the study area description in the revised manuscript as to your recommendation.

4. In the calculation of the sample size, the formula yields 376 not 260. If 10% contingency is used it will be 414. Rather, you can estimate the number of admissions based on a three-month data and then you can use a sample adjustment formula for finite population.

**Response:** Thank you reviewer for the input. We have recalculated the sample size and included it in the revised manuscript as to your recommendation. The sample size was determined using the sample size formula for incidence proportion with finite population correction for a population of less than 10,000 (N= 450 in our case as we admit nearly 25 patients monthly), and 10% contingency. With an expected incidence proportion of 57%(P) for MODS, a margin of error of 5%(e), a 95% confidence level(Z), and an 18-month (1.5 year) follow-up period(T) a sample size of 566 patients was calculated using the formula: **n_0_ = Z^2^ * P * (1-P) * T / e^2^**. Upon correction of this figure for a finite population using the formula: **n_f_ = n_0_/1+ (n_0_/N)** and 10% contingency added, the final sample size was found to be 276 patients. Though the calculated sample size was 276, we included 310 patients in the final analysis.

5. In the discussion section (paragraph 2, line 4), the justification you indicated has to be accompanied by references.

**Response:** Thank you reviewer for the recommendation. We have included citations in the revised document.

6. In the discussion section paragraph 3, the justification you raised on the increased risk of MODS in critically ill children who take traditional herbal medicines has to be supported by references.

**Response:** Thank you reviewer for the recommendation. We have included citations in the revised document.

7. In the introduction (on the last paragraph) and data processing and analysis part, avoid using first-person pronouns ("we").

**Response:** We have corrected it in the revised manuscript.

**Reviewer #2:**

Thank you, reviewer, for the general comments about the study. We have accepted the limitations of the study you considered and included them in the limitation part of the revised manuscript.

**Comments on title and abstract:**

1. Title not specific to study area: I will suggest that the authors revise the title to make it more specific to the study area. This could help readers quickly understand what the study is about and whether it is relevant to their interests.

**Response:** We have corrected it in the revised manuscript.

2. Lack of background of the study: I suggest that the authors include more information about the background of the study in the background section of the abstract. This could help readers understand the context of the research and why it is important.

**Response:** Thank you reviewer for the suggestion. We have included background information in the revised manuscript.

3. It is not explicitly stated in the abstract where the single center study took place. To improve the readers' understanding of the study's limitations and generalizability, I suggest that the authors include a sentence in the abstract specifying that the study was conducted at Gondar Comprehensive Specialized Hospital, Gondar, Ethiopia

**Response:** We have corrected it in the revised manuscript.

**Population and sample part (p.4):**

The sample size for the study appears to have been determined using a standard formula for incidence proportion with finite population correction, taking into account the expected incidence proportion of MODS, margin of error, confidence level, and follow-up period. However, there is some discrepancy in the reported sample size calculation, with the statement indicating a sample size of 260 patients, while later indicating that a total of 310 patients were included in the final analysis. Moreover, there is no explicit mention of sampling techniques

**Response:** We used a consecutive sampling technique as the study population size was too small (N=450 based on our monthly admission reports). The minimum sample size calculated was 276. But we included 310 patients in the final analysis, though it was above the calculated sample size, it will increase the representativeness of the findings of the study. We have made some amendments. Thank you.

**Variables of the study part (p.5)**

1. There is no explicit mention of a variable related to traditional herbal medicine (THM) use in the list of independent variables considered in the study.

**Response:** Thank you reviewer for the insight. It is corrected in the revised manuscript.

2. The authors of a study on Multiple Organ Dysfunction Syndrome (MODS) should include information about the criteria used for the diagnosis of MODS in their manuscript.

**Response:** We have mentioned it in the operational definition part of the document. “Event (MODS): In this study, MODS is defined as having physiologic derangement in two or more organ systems.”

**Data processing and analysis (p.6)**

The statistical methods used in the study should be reviewed to ensure that appropriate methods were used for the type of data analyzed. For analyzing time-to-event data, such as the time to development of MODS, survival analysis techniques such as bivariate and multivariate Cox proportional hazards models are appropriate, while the chi-square test and simple linear regression are not. Therefore, the chi-square test and simple linear regression should be replaced with appropriate statistical methods like bivariate and multivariate Cox proportional hazards models. However, it appears that the appropriate statistical methods were used in the results section of the manuscript.

**Response:** Dear Reviewer. We used appropriate statistical tests for survival analysis. We included linear regression and chi-square test in addition, only when it is found to be necessary. “……. summary statistics such as life-table, log-rank test, and Kaplan-Meier curves were computed to determine the incidence rate of MODS and compare survival curves between different categories of explanatory variables. Statistical tests such as the chi-square test and simple linear regression were used when they are found to be necessary. Finally, both bivariate and multivariate Cox proportional hazards models were used to identify the predictors of MODS.”

**Results part:**

1. As the objective of the study is to examine the incidence and predictors of Multi-Organ Dysfunction Syndrome (MODS) in a Pediatric Intensive Care Unit (PICU) in Ethiopia, the Cox proportional hazards model can be utilized to investigate the association between predictor variables and the likelihood of developing MODS over time, rather than the mortality of patients (p.10). Mortality of patients should be removed.

Response: We stated the incidence of MODS in the manuscript as follows. “The study monitored 310 ICU patients for a total of 1245 person-day observations (41.5 person-months) and found that 30.96% (95% CI: 25.8, 36.6) developed Multi-Organ Dysfunction Syndrome (MODS). Among those with MODS, 42.70% had it at admission or within 24 hours, while the remainder developed it during their ICU stay, resulting in an incidence rate of 7.71 (95% CI: 6.10, 9.40) per 100-person day observation. The median time to the development of MODS was 11.81 days.” We mentioned mortality as an additional outcome.

2. Patients who used THM had an increased risk of MODS AHR of 2.45 (95% CI: 1.29, 4.65). Therefore, a hazard ratio of 2.45 is slightly lower than a three-fold increase in risk. It will be better nearly three-fold.

**Response:** Thank you reviewer. We have made a correction in the revised manuscript. We stated it as “……TMH doubled the risk of MODS.”

**Regarding figures:**

Figures 3, 4, and 5 are likely to be important components of the study and should be properly described, labeled, and cited in the text to ensure that readers can fully understand and interpret the results presented. It is important that the author reviews the manuscript and ensures that figures are properly integrated into the text to support the study's findings and conclusions.

**Response:** Thank you reviewer. We have cited the figures in the appropriate parts of the revised manuscript.
