# Supplementary material for: Traditional herbal medicine use doubled the risk of multi-organ dysfunction syndrome in children: A prospective cohort study
Source: PLoS One. 2024 Feb 23;19(2):e0286233. doi: 10.1371/journal.pone.0286233 (PMC10889611; doi:10.1371/journal.pone.0286233)
Supplement: Attachment — Submitted filename: Response to the Reviewers.docx [file pone.0286233.s003.docx]

**Response to the Reviewers**

**Manuscript number:** PONE-D-23-14246R1

# **Title:** Traditional Herbal Medicine Use Doubled the Risk of Multi-organ Dysfunction Syndrome in Children in Gondar, Ethiopia: A Prospective Cohort Study.

Authors;

Nahom Worku Teshager: ([nahomworkarm@gmail.com](mailto:nahomworkarm@gmail.com))

Koku Sisay Tamirat: [kokusisay23@gmail.com](mailto:kokusisay23@gmail.com)

Ashenafi Tazebew Amare: [ashenafitazebew1@gmail.com](mailto:ashenafitazebew1@gmail.com)

Mulualem Endeshaw Zeleke: [muleaman45@gmail.com](mailto:muleaman45@gmail.com)

Asefa Admasu Taddese: [adimasuasefa@gmail.com](mailto:adimasuasefa@gmail.com)

We are grateful for the helpful feedback and constructive suggestions by the editors and reviewers that helped us to improve the quality of the manuscript. We also appreciate your acknowledgment of the fact that this is a very important study describing the role of traditional Herbal Medicine intake in the risk of development of MODS. This study tried to show how traditional herbal medicine intake affects the incidence of MODS among pediatric age groups. We carefully responded to all points raised by the editorial board and reviewers and have modified the manuscript accordingly. We have submitted the data as a supporting file as we have no legal restrictions. Since all authors are from a developing country (Ethiopia), we are eligible for waiver of the article processing charge. We hope you will consider our work for publication.

With regards!

Authors.

**Response to Editorial Board:**

Dear editorial Boar,

We have amended the manuscript as to the recommendations and comments you provided us. Thank you for contributing for the improvement of our work. We hope we have addressed all the issues/points you stated.

1. The Title is too long.  Please revise it.

Response: Thank you for the suggestion. The title is revised. I ,previously, included the institution name as per the recommendation of one of the reviewers.

2. Please review your reference list to ensure that it is complete and correct.

Response: we have reviewed and made sure the references are correct.

**Response to Reviewer #1**

1. In the abstract section before the last sentence, "A threefold increase in the risk of MODS....) has to be stated consistently as " A more than twofold increase in the risk of MODS was seen in patients who used TMH".

**Response:** Thank you, reviewer, for the input. We have corrected it in the revised manuscript as to your recommendation.

2. In the competing interest section, it is stated that "---patients/public were not involved in the research". However, the study is a prospective cohort and needs patient’s follow-up. How it is said that patients were not involved? **Response:** Thank you for bringing to my attention the apparent contradiction in the competing interest section. By "---patients/public were not involved in the research “, we mean that patients/public are not directly involved in the design and data analysis of the research. We have modified it as “There were no competing interests, patients and/or public were not directly involved in the design, conduct, or reporting of the research.”
3. In the data processing and analysis section, the first sentence has to be edited as of the following. "After ensuring consistency and completeness, the data was imported into EpiData V.3.1, then exported to Excel and subsequently to R 4.2.2 for cleaning and analysis."

**Response:** Thank you, reviewer. Done as to your recommendation.

4. In the data processing and analysis section, the authors has to replace the term multivariate by Multivariable, since the study has only one dichotomous outcome.

**Response:** Thank you, reviewer. Corrected as to your recommendation.
