# Supplementary material for: Traditional herbal medicine use doubled the risk of multi-organ dysfunction syndrome in children: A prospective cohort study
Source: PLoS One. 2024 Feb 23;19(2):e0286233. doi: 10.1371/journal.pone.0286233 (PMC10889611; doi:10.1371/journal.pone.0286233)
Supplement: Attachment — Submitted filename: Response to the Reviewers.docx [file pone.0286233.s004.docx]

**Response to the Reviewers**

**Manuscript number:** PONE-D-23-14246R2

# **Title:** Traditional Herbal Medicine Use Doubled the Risk of Multi-organ Dysfunction Syndrome in Children: A Prospective Cohort Study.

Authors;

Nahom Worku Teshager: ([nahomworkarm@gmail.com](mailto:nahomworkarm@gmail.com))

Koku Sisay Tamirat: [kokusisay23@gmail.com](mailto:kokusisay23@gmail.com)

Ashenafi Tazebew Amare: [ashenafitazebew1@gmail.com](mailto:ashenafitazebew1@gmail.com)

Mulualem Endeshaw Zeleke: [muleaman45@gmail.com](mailto:muleaman45@gmail.com)

Asefa Admasu Taddese: [adimasuasefa@gmail.com](mailto:adimasuasefa@gmail.com)

We are grateful for the helpful feedback and constructive suggestions by the editors and reviewers that helped us to improve the quality of the manuscript. We also appreciate your acknowledgment of the fact that this is a very important study describing the role of traditional Herbal Medicine intake in the risk of development of MODS. This study tried to show how traditional herbal medicine intake affects the incidence of MODS among pediatric age groups. We carefully responded to all points raised by the editorial board and reviewers and have modified the manuscript accordingly. We have submitted the data as a supporting file as we have no legal restrictions. Since all authors are from a developing country (Ethiopia), we are eligible for waiver of the article processing charge. We hope you will consider our work for publication.

With regards!

Authors.

**Response to Editorial Board:**

Dear editorial Boar,

We have amended the manuscript as to the recommendations and comments you provided us. Thank you for contributing for the improvement of our work. We hope we have addressed all the issues/points you stated.

1. Is it necessary to specify the location in the Title?

**Response:** Thank you for the suggestion. The title is revised.

2. Journal Requirements: References

Response: I have checked my citations. All the articles I cited are available online. I checked them one by one on google scholar.

**Response to Reviewer #2**

1. In the Data processing and analysis section, the statement "Statistical tests such as the chi-square test and simple linear regression were used when they are found to be necessary" should be removed. These tests are not appropriate for survival data analysis in this study, and the statement is inaccurate. Instead, please include only the methods that were actually employed for analysis

**Response:** Thank you, reviewer, for the input. We have corrected it in the revised manuscript as to your recommendation. As this is survival analysis, we used KM curve, log rank test and Cox PH model.

2. The section currently titled “Predictors of Mortality in the PICU” should be renamed to “Predictors of Incidence of MODS in the PICU”. Furthermore, all instances of the term “mortality” should be replaced with “incidence of MODS” in this section. This is because the primary objective of this study is to investigate the incidence and predictors of MODS in a PICU, not mortality. Additionally, please note that Table 2 does not discuss mortality.”

**Response:** Thank you, reviewer, that was unseen mistake on our side. We have corrected it in the revised manuscript as to your recommendation. Regarding table 2, it is a regression table for explanatory variables of MODS. On the footnote, we put the long form of mPIM 2 : modified pediatric mortality score 2 which we used to assess severity of illness at admission.

3. In the Discussion section, the statement “The finding that patients with critical illness diagnoses had a 2.45 times increased risk of developing MODS in critically ill patients is supported by multiple other studies” should be corrected. The correct value is not 2.45, but rather 2.68.

**Response:** Thank you. Corrected in the revised manuscript.

4. In the Author Contributions section, the roles of the authors need to be clearly defined.

**Response:** Thank you, reviewer. We have clearly put the authors’ contribution in the revised manuscript
